# Antimalarial Properties of Isoquinoline Derivative from *Streptomyces hygroscopicus* subsp. Hygroscopicus: An In Silico Approach

**DOI:** 10.1155/2020/6135696

**Published:** 2020-01-08

**Authors:** Rivo YB. Nugraha, Icha FD. Faratisha, Kana Mardhiyyah, Dio G. Ariel, Fitria F. Putri, Nafisatuzzamrudah, Sri Winarsih, Teguh W. Sardjono, Loeki E. Fitri

**Affiliations:** ^1^Department of Parasitology, Faculty of Medicine, Universitas Brawijaya, 65145 Malang, East Java, Indonesia; ^2^Malaria Research Group, Faculty of Medicine, Universitas Brawijaya, Universitas Brawijaya, Malang 65141, East Java, Indonesia; ^3^Study Program of Medicine, Faculty of Medicine, Universitas Brawijaya, 65145 Malang, East Java, Indonesia; ^4^Department of Biochemistry & Biomolecular, Faculty of Medicine, Universitas Brawijaya, 65145 Malang, East Java, Indonesia; ^5^Doctoral Program in Medical Sciences, Faculty of Medicine, Universitas Brawijaya, 65145 Malang, East Java, Indonesia; ^6^Study Program of Pharmacy, Faculty of Medicine, Universitas Brawijaya, Malang 65141, East Java, Indonesia; ^7^Master Program in Biomedical Science, Faculty of Medicine, Universitas Brawijaya, Malang 65141, East Java, Indonesia; ^8^Department of Microbiology, Faculty of Medicine, Universitas Brawijaya, Malang 65141, East Java, Indonesia

## Abstract

Malaria is one of the life-threatening diseases in the world. The spread of resistance to antimalarial drugs is a major challenge, and resistance to artemisinin has been reported in the Southeast Asian region. In the previous study, the active compound of *Streptomyces hygroscopicus* subsp. Hygroscopicus (*S*. *hygroscopicus*), eponemycin, has been shown to have antimalarial effects. To further analyze the effects of other active compounds on the *Plasmodium* parasite, identifying and analyzing the effectiveness of compounds contained in *S*. *hygroscopicus* through instrumentation of liquid chromatography/mass spectrometry (LC/MS) and in silico studies were very useful. This study aimed at identifying other derivative compounds from *S*. *hygroscopicus* and screening the antimalarial activity of the compound by assessing the binding affinity, pharmacokinetic profile, and bond interaction. The derivative compounds were identified using LC/MS. Protein targets for derivative compounds were found through literature studies, and the results of identification of compounds and protein targets were reconstructed into three-dimensional models. Prediction of pharmacokinetic profiles was carried out using Swiss ADME. Screening of protein targets for the derivative compound was carried out using the reverse molecular docking method. Analyzing bond interaction was done by LigPlot. One compound from *S*. *hygroscopicus*, i.e., 6,7-dinitro-2-[1, 2, 4]triazole-4-yl-benzo[de]isoquinoline-1,3-dione, was successfully identified using LC/MS. This compound was an isoquinoline derivative compound. Through literature studies with inclusion criteria, thirteen protein targets were obtained for reverse molecular docking. This isoquinoline derivative had the potential to bind to each protein target. The pharmacokinetic profile showed that this compound had the drug-likeness criteria. *Conclusion*. 6,7-Dinitro-2-[1, 2, 4]triazole-4-yl-benzo[de]isoquinoline-1,3-dione has antimalarial activity as shown by reverse molecular docking studies and pharmacokinetic profiles. The best inhibitory ability of compounds based on bond affinity is with adenylosuccinate synthetase.

## 1. Introduction

Malaria is one of the top ten deadly diseases in the world and has been a global health threat for centuries [1]. At present, malaria is estimated to be endemic in 91 countries and one of them is Indonesia [2]. The incidence of malaria cases or Annual Parasite Incidence (API) in Indonesia in 2011 to 2015 continued to decline from 1.75% to 0.85% per 1000 population. The API number reaches zero or malaria-free in several regions, such as DKI Jakarta, West Java, East Java, Banten, and Bali. However, most parts of Eastern Indonesia still have high API numbers, one of which reaches 31.93% in the Papua region [3].

Facing malaria is a big challenge because of the presence of antimalarial drug resistance [4]. The endemic countries have changed therapeutic policies, from monotherapy to combination therapy, artemisinin-based combination therapies (ACT), but artemisinin resistance in *P*. *falciparum* in Southeast Asia has been reported [5]. In addition, resistance to antimalarial drugs such as chloroquine and sulfadoxine-pyrimethamine has also been noted, especially in malaria-endemic areas. Other antimalarial drugs such as mefloquine, halofantrine, atovaquone, proguanil, artemether, and lumefantrine have good efficacy, but there are limitations such as cost [6].

The emergence of resistance to antimalarial drugs has become an urgent need to develop effective new antimalarial compounds. The process of finding a drug starts from the identification of unmet medical needs, it is the condition in which there is no satisfaction with the method of diagnosis, therapy, and prevention, and then proceeds with the identification of biological targets for drug-able targets. Protein targets that are compatible with the drug or compound will improve the symptoms of the disease or have a relation to the causative process of the disease [6]. In this revolution era of molecular biology, there are a lot of protein targets that had been successfully identified in *Plasmodium*, such as plasmepsins, falcipains, proteases, peptidases, reductoisomerases, purine nucleoside phosphorylase, thymidylate synthase, pyruvate kinase, and dihydrofolate reductase [6]. The known protein targets lead to clear understanding about the mechanism of action of the antimalarial target towards each target. The prior known antimalarial also has different pathways towards *Plasmodium*; quinoline derivatives act by accumulation of food vacuoles in parasites, inhibition of heme detoxication, and inhibition of respiration reaction of parasites in cytochrome BC1 complex. Antifolate derivatives have another pathway; they inhibit dihydropteroate synthetase (PfDHPS), biosynthesis of folic acid, and dihydrofolate reductase (PfDHFR). Antibiotics such as tetracycline, doxycycline, and clindamycin have antimalarial effect by inhibiting protein synthetase pathway and preventing the binding of aminoacyl-tRNA to mRNA ribosome complex. However, there are several antimalarial drugs that have unclear mechanisms such as artemisinin derivatives and primaquine as quinoline derivative [7].

The use of natural products as a therapy for the disease has been carried out since several years ago because it can produce certain biological activities and has properties such as a drug [8]. One proof of the successful development of drugs from natural products is the discovery of artemisinin and quinine. These drugs have been used extensively as antimalarial therapy [9]. One study conducted that utilized natural product is *Streptomyces hygroscopicus* subsp. *hygroscopicus* (*S*. *hygroscopicus*), by extracting the active fraction of bacterial secondary metabolites. The active fraction of this bacterium has an antimalarial activity with the protein target the ubiquitin-proteasome system (UPS) [10]. The active fraction of the *S*. *hygroscopicus*, eponemycin, is a proteasome inhibitor that can inhibit the function of the UPS in eukaryotic cells, including *Plasmodium*. Analysis of eponemycin analogues from the metabolite extract of *S*. *hygroscopicus* has also been carried out by another research by Fitri et al. [11] who used the thin-layer chromatography (TLC) and high-performance liquid chromatography (HPLC) methods and stated that the active fraction of *S*. *hygroscopicus* has the potential to be a candidate for new antimalarial drugs and can cause stress on *Plasmodium berghei* [11].

The process of further analysis and identification of other derivative compounds of *S*. *hygroscopicus* is very necessary to find out more details about the profile and potential of the derivative compound from *S*. *hygroscopicus* as an antimalarial agent. In silico study as a visual screening method is one of the choices in the drug discovery process. In silico method aims at predicting the orientation of molecular bonds (ligands) with other molecules (protein targets) to form a stable complex [12]. In silico approach is often used in the process of discovering new drugs because it is considered to have many advantages in terms of cost, time efficiency, and work effectiveness [13]. The main focus of the in silico is docking, which is the program used to predict the accuracy of the bonding mode of the protein-ligand complex through the conformation and scoring stages. Docking is usually used to predict various types of ligands for one specific protein target. There is another method known as reverse docking. In contrast to docking, reverse docking is used to predict the bonding of one ligand with various protein targets. Reverse docking is usually used to find out new targets of a drug whose mechanism of action is known or natural products have unknown therapeutic effects [14]. Reverse docking in this study can be used to determine and assess the potential of the active compound of *S*. *hygroscopicus* as an antimalarial agent through its association with protein targets in *Plasmodium* parasite.

Based on the above, we report the newest antimalarial agent, the isoquinoline derivative from *S*. *hygroscopicus* through in silico approach.

## 2. Materials and Methods

### 2.1. Design and Settings

The design of the study was explorative method using LC/MS and continued to reveal the antimalarial activities of the compound in silico through protein-ligand interactions in reverse molecular docking. This research was conducted from September to November 2018. *S*. *hygroscopicus* culture was handled in the Laboratory of Microbiology, Faculty of Medicine, Universitas Brawijaya. *S*. *hygroscopicus* extraction was carried out at Laboratory of Pharmacy, Faculty of Medicine, Universitas Brawijaya. LC/MS analysis activities were carried out at PT. Angler Biochemlab, Surabaya, and the in silico study was carried out together with Inbio Indonesia.

### 2.2. Culture of *Streptomyces hygroscopicus* subsp. *hygroscopicus*

Bacteria *S*. *hygroscopicus* was obtained from LIPI Microbial Collection, Cibinong, Indonesia. The colony *S*. *hygroscopicus* then confirmed its characterization based on the macroscopic colony, morphology, and microscopic staining of Gram. It is Gram-positive bacteria, has endospore, and produces hyphae.

### 2.3. Secondary Metabolic Fermentation of *Streptomyces hygroscopicus* subsp. *hygroscopicus*

Fermentation using ISP4 liquid was in the amount of 50 ml under aseptic conditions in 250 ml Erlenmeyer flask. Bacterial colonies were scraped from ISP4 culture medium and homogenized in ISP4 broth media. It was incubated at 28°C on a 150 rpm shaking incubator for 5 days. After that, the inoculum for the fermentation process was ready to use. 100 ml of 7.0–7.4 adjusted pH ISP4 broth was autoclaved. Next, 25.8 × 10^6^ bacteria from inoculum were added to the broth and fermented at 28°C with 150 rpm of shaking incubator for 5 days [10, 11].

### 2.4. Extraction Procedure for Metabolites of *Streptomyces hygroscopicus* subsp. *hygroscopicus*

After fermentation, the media were harvested and centrifuged to remove cells and debris. The filtrate was collected in a separating funnel. The fermented broth was filtered. The filtrate was mixed with an ethyl acetate ratio of 1 : 1 (v/v) and hand-shaken for 1 hour in a separating funnel. The solvent phase containing metabolic compounds was separated from the aqueous phase. The solvent phase was evaporated to dryness in a water bath.

### 2.5. Liquid Chromatography/Mass Spectrometry (LC/MS) Analysis

Crude extract samples from *S*. *hygroscopicus* were injected and analyzed by the UPLC and ultra high-resolution time-of-flight mass spectrometry (TOF-MS) detector in cooperation with PT. Angler Indonesia, Surabaya.

### 2.6. Pharmacokinetic Profile

The derivative compound of *S*. *hygroscopicus* was analyzed by pharmacokinetic profile using Swiss ADME by entering the SMILES formula.

### 2.7. Construction of Protein Target Database

In this study, we obtained protein target database through the literature studies and Therapeutic Target Database (TTD) [6, 15, 16]. The protein target screening processes were done by searching using the keywords “isoquinoline,” “malaria,” and “protein target.” We selected and downloaded the protein targets that fulfil the criteria in Protein Data Bank via the http://www.rscb.organd can be downloaded as an extension file .pdb. The protein targets had to fulfil the criteria: (1) they have significant roles in *Plasmodium* survival and their inhibition may cause parasite death, (2) the protein targets have their own native ligands, (3) the native ligands could not be peptide or protein, and (4) it has been docking to other isoquinoline compounds related to malaria disease (Table 1). Then, we did the residual separation and optimization of protein target. Optimization involved removing the remaining water, ligands from the crystallization process, and cofactors with the PyMol 2.0 program. The residue that is still in the protein will affect the bond between the protein and the ligand, so it needs to be separated first so that the docking results can be optimal. The results of this process were saved in the form of .pdb.

### 2.8. Preparation of Ligands and Target Structures

The ligand structure was prepared. The ligand was obtained from the identification of the derivative compound of *S*. *hygroscopicus* via LC/MS. The derivative compound had been identified and its chemical structure was manually drawn with MarvinSketch and downloaded in the form of .sdf or .pdb. The information of native ligands for their protein target was found in RSCB Protein Data Bank, and the 3D structure was downloaded from PubChem as .sdf file, and another native ligand was taken from cryocrystallized ligand in each protein target and saved as .pdb file.

### 2.9. Reverse Molecular Docking Studies

Reverse molecular docking was done using PyRx 0.9.5, i.e., a visual screening software for computational drug discovery that could be used in various chemical compounds against potential drug targets [44], with specific dimension of grid box based on native ligands' protein targets (Table 2). We had docked each protein target with its native ligand and the derivative compound of *S*. *hygroscopicus* three to five times for accuracy. The results were binding affinity scores between the ligands and the protein targets. After that, the binding affinities were compared between the ligand from *S*. *hygroscopicus* and protein targets' native ligands to each protein target. Docking results were visualized using Chimera 1.13.1 program.

### 2.10. Visualization of Interaction

The stronger binding affinities between the derivative compound and protein targets compared to the native ligands were visualized using LigPlot 1.4.5. The visualization is used to assess whether the residues that formed are consistent with the score of binding affinities [14]. The derivative compound is predicted to have strong bond to the targets if it is capable of binding strongly through hydrogen bonds with the same amino acid residue compared to the control ligand [45].

## 3. Results and Discussion

### 3.1. LC/MS Analysis

The purpose of identifying the compound of *S*. *hygroscopicus* using the LC/MS analysis method was to find out the type of the compound in *S*. *hygroscopicus*. Analysis was done by looking at mass-to-charge ratio (*m*/*z*) with the percentage of abundance (% abundance) or intensity. In addition, in the total ion chromatogram (TIC), derivative compounds could be observed based on molecular weight and certain retention time (RT) peaks. The molecular weight of time would be compared with the LC/MS database so that the name of the active compound can be known (Table 3).

**Figure 1:**
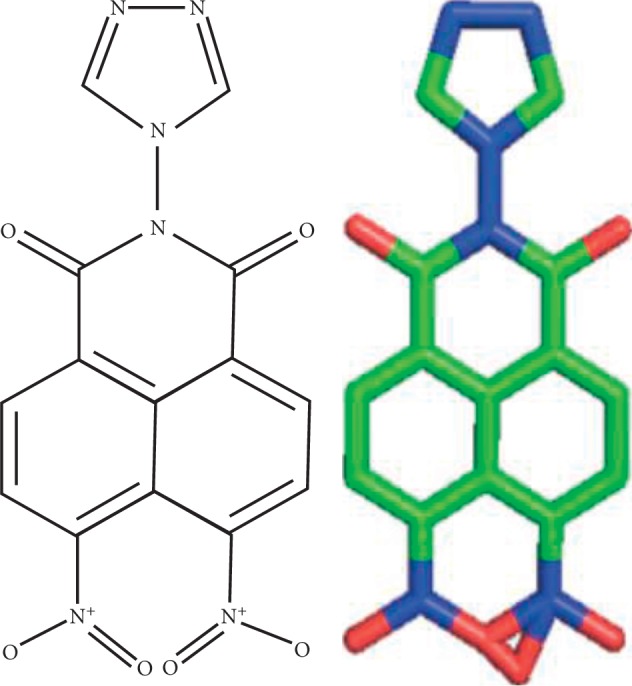
Chemical structure of 6,7-dinitro-2-[1, 2, 4]triazole-4-yl-benzo[de]isoquinoline-1,3-dione.

The results of the LC/MS analysis showed several peaks of the compounds. Each peak was identified based on the molecular weight and fragments, and the results of the identification of the five highest peaks were obtained. Of the five highest peaks analyzed by RT 0–15 minutes, there was one identifiable compound which is 6,7-dinitro-2-[1, 2, 4]triazole-4-yl-benzo[de]isoquinoline-1,3-dione with a molecular weight of 355.0422 kDa, while four other compounds have not been identified. The ligand was prepared for its structure. It was obtained from the depiction in MarvinSketch (Figure 1). SMILES formula of ligand, c1cc2c(=O)n(c(=O)c3c2c(c1[N+](=O)[O-])c(cc3)[N+](=O)[O-])n1cnnc1, was done by using Open Babel. The ligand structure was then converted to the .pdb format.

6,7-Dinitro-2-[1, 2, 4]triazole-4-yl-benzo[de]isoquinoline-1,3-dione has the chemical formula C_14_H_7_N_6_O_6_^+1^ at 3.77 minutes, and it produces two ion fragments, namely, C_6_HN_6_O_2_^+1^ and C_8_H_7_O_4_^+1^. 6,7-Dinitro-2-[1, 2, 4]triazole-4-yl-benzo[de]isoquinoline-1,3-dione is one of the derivatives of isoquinoline. Isoquinoline is a heterocyclic organic aromatic compound, isomer with quinoline. The term isoquinoline is used to make references to isoquinoline derivatives. For example, benzylisoquinoline is a compound with a backbone structure naturally similar to isoquinoline and also papaverine and morphine. Cellular location of isoquinoline is in the cytoplasm [46]. Isoquinoline and its derivatives have been widely used as therapeutic agents for atherosclerosis therapy [47] and antibacterial [48], antituberculosis [49], anticancer [50], and antimalarial [51, 52] agents, and others.

In the previous study, the active compound of the secondary metabolite of *S*. *hygroscopicus*, eponemycin, is known to have antimalarial effects through inhibition of parasitic UPS [10, 11]. Another compound that was identified in this study was 6,7-dinitro-2-[1, 2, 4]triazole-4-yl-benzo[de]isoquinoline-1,3-dione; this compound is a derivative of isoquinoline. Previous studies have suggested that isoquinoline and its derivatives have antimalarial effects, for example, isoquinoline alkaloids and 10-demethylxylopinine, which originate from *Actinodaphne macrophylla* [51], protopine and coreximine from *Corydalis crispa*, and crude alkaloid extracts from Brazilian plant species [52].

### 3.2. Prediction of Pharmacokinetic Profile

The pharmacokinetic profile of a compound greatly influences the effectiveness of drugs in the body. Pharmacokinetic describes how the body treats drugs or compounds by looking at the processes of absorption, distribution, metabolism, and excretion (ADME) [53]. To find out the effectiveness of compound drugs that are good in the oral route administration, there are several criteria that must be met. One method of assessment is the Rule of Five from Lipinski et al. [54], which includes the following: (1) the molecular weight of the compound is less than 500 mg/dL; (2) a lipophilicity (clogP) is less than 5; (3) hydrogen bond donor is less than 5; and (4) hydrogen bond acceptor is less than 10.

Based on Table 4, this isoquinoline derivative is complied with Lipinski's criteria, molecular weight 354.23 g/mol, 8 H-bond acceptor, 0 H-bond donor, and clogP of 0.08. These results indicated that isoquinoline derivative compounds can be administered via the oral route. cLogP or lipophilicity of isoquinoline derivative compounds is 0.08 which means of having a good lipophilicity (< 5). Good lipophilicity will facilitate compounds in the absorption process, not released by albumin easily, so that the toxicity effects can be reduced and they can be rapidly metabolized and slightly excreted in the kidneys [55].

### 3.3. Reverse Molecular Docking Results

**Figure 2:**
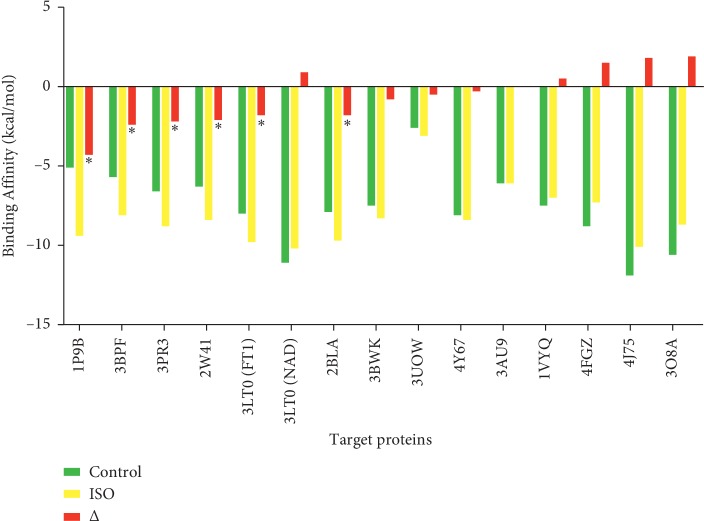
The result of reverse molecular docking. Binding affinities of control ligands toward each protein target (green bars) are compared with binding affinities of isoquinoline (ISO) toward same protein targets. Red bars show binding affinity differences between the two ligands. Asterisk indicates the highest and most negative binding affinity differences.

The reverse molecular docking results in Figure 2 showed that 6,7-dinitro-2-[1, 2, 4]triazole-4-yl-benzo[de]isoquinoline-1,3-dione can bind to protein targets with various binding affinities compared to each native ligand of protein targets. The binding affinity is the ability of ligands to bind to the protein target; the more negative the binding affinity between the ligand and the protein, the better the binding so that the compound can have a biological activity on the protein target [56]. The results of reverse molecular docking between the isoquinoline derivatives with fourteen protein targets showed that the isoquinoline derivative has different binding affinities with each of the protein targets compared to the native ligands. There are eight protein targets (1P9B, 3BPF, 3PR3, 2W41, 2BLA, 3BWK, 3UOW, and 4Y67) that have more negative values than the native ligands, four protein targets (1VYQ, 4FGZ, 3O8A, and 4J75) that have more positive value than the native ligands, one protein target (3AU9) that has same binding affinity, and one protein target (3LT0) that has more positive and negative values compared to two different native ligands.

In order to identify specific protein target for which the isoquinoline derivative compound can be therapeutic, based on the most negative delta in Figure 2, there are six protein targets that potentially have stronger interaction than the native ligands based on the binding affinities.

The strongest binding affinity of this compound to the protein target was a bond with the adenylosuccinate synthetase (PDB ID: 1P9B) compared to its native ligand, hadacidin, as shown in Figure 2. The binding affinity between the isoquinoline derivative and adenylosuccinate synthetase is more negative, which is −9.4 kcal/mol, while the binding affinity between the control ligand (hadacidin) and a adenylosuccinate synthetase is −5.1 kcal/mol. The difference in the binding affinity of isoquinoline derivatives with hadacidin is −4.3 kcal/mol; this value is the highest difference between the isoquinoline derivative compound ligand and other native ligands of protein target.

Adenylosuccinate synthetase plays an important role in purine biosynthesis in both de novo pathways and salvage pathways [57]. This enzyme catalyzes the reaction of adenylosuccinate formation from IMP and aspartate in the salvage pathway from hypoxanthine [57, 58]. Inhibition of adenylosuccinate synthetase will affect the balance of the AMP/GMP ratio in cells and lead to death of the parasites [57, 58]. Hadacidin as a native ligand compared to the compound 6,7-dinitro-2-[1, 2, 4]triazole-4-yl-benzo[de]isoquinoline-1,3-dione inhibits the adenylosuccinate synthetase through competition with aspartate. Hadacidin is an analogous metabolite of L-aspartate and becomes a potent inhibitor of adenylosuccinate synthetase, thus inhibiting the synthesis of AMP [59–61].

Adenylosuccinate synthetase in *Plasmodium falciparum* (*Pf*AdSS) has kinetic resemblance to acidic isozyme from mice whose main function is to carry out purine biosynthesis, whereas in other species such as *E*. *coli* and humans, they show several characteristic differences [61]: (1) In *P*. *falciparum*, the content of A/T (adenine/thymine) is higher (>70%) than in *E*. *coli* and mice, so it takes 3-4 times more adenine (AMP precursors) than the number of guanine (GMP precursors). Adenylosuccinate synthetase regulation in maintaining the AMP/GMP ratio in *Plasmodium* cells has a very important role because of this difference in A/T levels; (2) the absence of the de novo pathway in *Plasmodium* makes adenylosuccinate synthetase the only source of AMP biosynthesis; (3) the bond sequence between adenylosuccinate synthetase and the other three substrates (IMP, guanosine triphosphate/GTP, and aspartate) to form adenylosuccinate and GDP (guanosine diphosphate) is different from other species. On *Pf*AdSS, the sequence is important, starting with the binding of IMP to adenylosuccinate synthetase, followed by GTP, followed by bonding with aspartate, whereas in other species the sequence of bonds tends to be random; (4) transfer of phosphoryl groups from GTP to IMP in *E*. *coli* enzymes and mice can occur in the absence of aspartate, but in *P*. *falciparum*, the transfer requires aspartate assistance; and (5) fructose-1,6-biphosphate (F16B) is a potent inhibitor of the adenylosuccinate synthetase in humans, whereas in *Plasmodium* this compound is an activator of the adenylosuccinate synthetase enzyme, especially in situations where GTP is limited.

**Figure 3:**
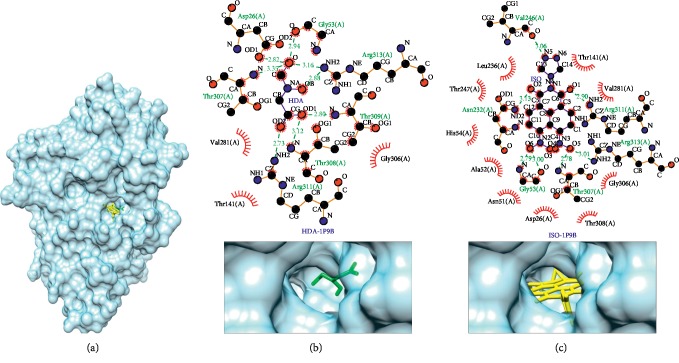
Binding complex and interaction visualization between adenylosuccinate synthetase (1P9B, blue), hadacidin (HDA, green), and isoquinoline derivative (ISO, yellow). In the ISO-HDA-1P9B binding complex (a), HDA and ISO share the same pocket. Interaction visualization of HDA-1P9B (b) shows it shares some similar residue interactions with ISO-1P9B (c).

The results of virtual screening using LigPlot showed the interaction of the compound 6,7-dinitro-2-[1, 2, 4]triazole-4-yl-benzo[de]isoquinoline-1,3-dione with the adenylosuccinate synthetase. This compound has 6 hydrogen bonds and 10 hydrophobic interactions while the native ligand (hadacidin) has 7 hydrogen bonds and 3 hydrophobic interactions, as shown in Table 5 and Figures 3(b) and 3(c). There are 9 same bonds between the compound–1P9B interaction and the control ligand–1P9B interaction. The hydrogen bonds that formed in compound–1P9B interaction shared the same amino acid residues compared to the control ligand, these were Arg311(A), Arg313(A), Thr307(A), and Gly53(A). Besides, the compound-1P9B's hydrophobic interactions shared several same residues as well compared to the control, these were Thr141(A), Val281(A), and Gly306(A). For the same amino acid residues involved, some had different types of bonds, these were Asp26(A) and Thr308(A). In 6,7-dinitro-2-[1, 2, 4]triazole-4-yl-benzo[de]isoquinoline-1,3-dione hydrophobic interactions are formed, while in the hadacidin, hydrogen bonds are formed. Raman et al. [57] stated that hadacidin binds to AdSS on Thr307 and the bond formed is a hydrogen bond. Isoquinoline derivative is also able to bind to the Thr307 amino acid from adenylosuccinate synthetase. Based on these data, the bonding interactions between the compound and protein target indicate that the compounds are able to work on the same side of the protein target and are able to interact better than the control assessed based on the number of bonds formed and the number of bond similarities compared to its native ligand.

**Figure 4:**
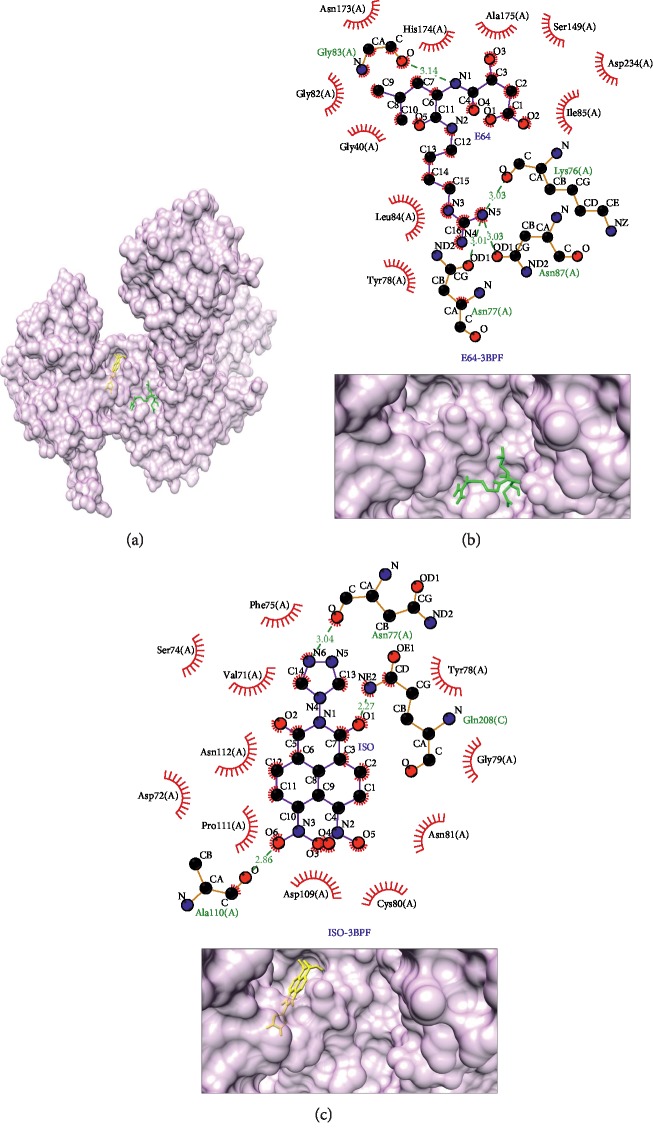
Binding complex and interaction visualization between falcipain 2 (3BPF, light purple), hydroxycarboxyethyl-carbonyl [leucylamino-butyl]-guanindine (E64, green), and isoquinoline derivative (ISO, yellow). In the ISO-E64-3BPF binding complex (a), E64 and ISO share the different binding pockets. Interaction visualization of E64-3BPF (b) shows mostly different residue interactions with ISO-3BPF (c).

The next most potential protein target was falcipain 2 (PDB ID: 3BPF, shown in Figure 4). The binding affinity between the isoquinoline derivative compound is −8.1 kcal/mol. It was stronger than the native ligand which is −5.7 kcal/mol. Falcipain 2 is known as the cysteine protease enzyme of *Plasmodium falciparum*. The role of falcipain 2 is to degrade haemoglobin in the trophozoite phase and to cleave the ankyrin and protein 4.1, i.e., an element of cytoskeleton for stabilizing red blood cell membrane in schizont phase [40, 62]. There were two same interaction bonds between isoquinoline derivative compound compared to the native ligand of falcipain 2, Asn77(A) as hydrogen bond and Tyr78(A) as hydrophobic interaction, as shown in Table 5 and Figure 4.

**Figure 5:**
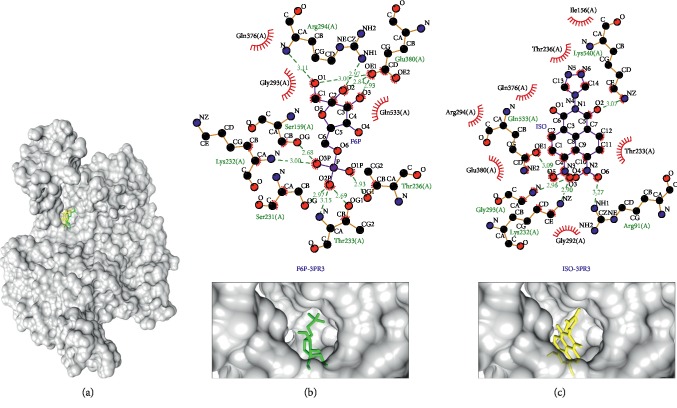
Binding complex and interaction visualization between glucose-6-phosphate isomerase (3PR3, grey), fructose-6-phosphate (F6P, green), and isoquinoline derivative (ISO, yellow). In the ISO-F6P-3PR3 binding complex (a), F6P and ISO share the same binding pocket. Interaction visualization of F6P-3PR3 (b) shows it shares some similar residue interactions with ISO-3PR3 (c).

Glucose-6-phosphate isomerase (PDB ID: 3PR3), as shown in Figure 5, was the third in the list of potential protein targets for the isoquinoline derivative compound (−8.8 kcal/mol) compared to its native ligand (−6.6 kcal/mol). The native ligand and the isoquinoline derivative compound shared several same-interacted residues, these were Lys232(A) as hydrogen bond and Gln376(A) as hydrophobic interaction. The remaining same interacted residues, i.e., Gln533(A), Thr236(A), Thr233(A), Gly293(A), Arg294(A), and Glu380(A), had different types of bonds, as shown in Table 5 and Figures 5(b) and 5(c). Glucose-6-phosphate isomerase is a key enzyme in glucose metabolism, i.e., glycolysis and gluconeogenesis. It catalyzes the second step of glycolysis, glucose-6-phosphate to fructose-6-phosphate [63–65]. This enzyme not only is a housekeeping enzyme of the sugar metabolism inside the cell, but also plays a role in various properties of cytokines and several other protein targets outside the cell [64]. Since *Plasmodium falciparum* use glucose as the major substrate of energy, this enzyme can be the potential target for antimalarial drugs [65].

**Figure 6:**
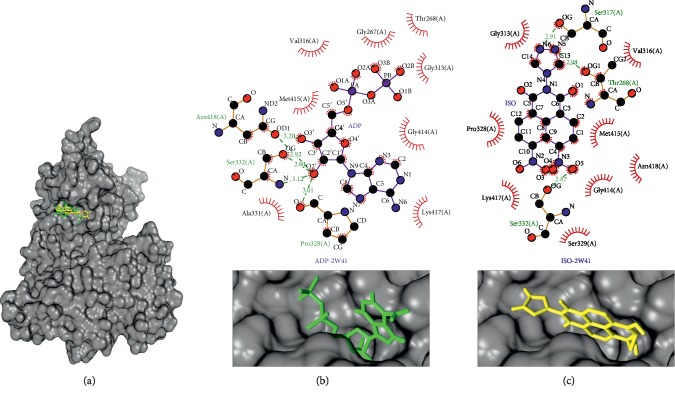
Binding complex and interaction visualization between glycerol kinase (2W41, black), adenine diphosphate (ADP, green), and isoquinoline derivative (ISO, yellow). In the ISO-ADP-2W41 binding complex (a), ADP and ISO share the same binding pocket. Interaction visualization of ADP-2W41 (b) shows it shares some similar residue interactions with ISO-2W41 (c).

Glycerol kinase (PDB ID: 2W41), as shown in Figure 6, interacts with the isoquinoline derivative compound at −8.4 kcal/mol and its native ligand at −6.3 kcal/mol. This protein target plays a role in the catalyzation of utilization of glycerol in plants and animals. It is very important for the carbohydrate and lipid metabolisms [28]. Glycerol kinase is the second most highly upregulated gene in *Plasmodium falciparum* gametocytes, with its expression not detectable in the asexual blood stage. Deletion of this gene had no effect on asexual and sexual (exflagellation) parasite growth. In kinetic studies, *Plasmodium falciparum* glycerol kinase is not regulated by fructose-1,6-biphosphate [28]. The interaction with this protein occurred with hydrogen bonds at Ser332(A) and hydrophobic interactions at Val316(A), Gly313(A), Gly414(A), Met415(A), and Lys417(A). The different types of interactions occur in the hydrogen bond Thr268(A) of the isoquinoline derivative compound; meanwhile, this bond in the native ligand's protein target is the hydrophobic interaction. Respectively, in the isoquinoline derivative compound, Pro328(A) and Asn418(A) formed hydrophobic interactions, but they formed hydrogen bonds in the native ligand, as shown in Table 5 and Figures 6(b) and 6(c).

**Figure 7:**
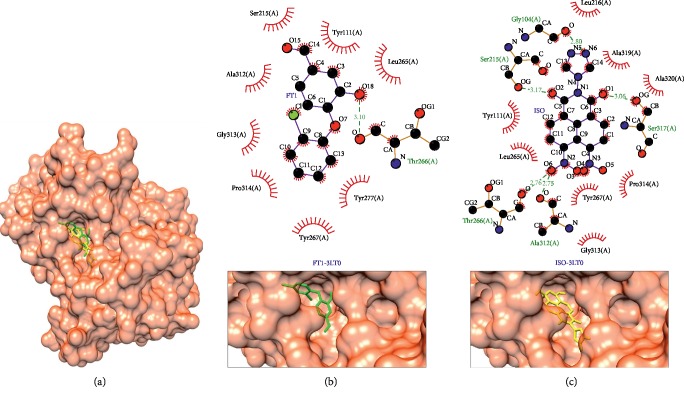
Binding complex and interaction visualization between enoyl-acyl carrier protein reductase (3LT0, orange), 4-(2,4-dichlorophenoxy)-3-hydroxybenzaldehyde (FT1, green), and isoquinoline derivative (ISO, yellow). In the ISO-FT1-3LT0 binding complex (a), FT1 and ISO share the same binding pocket. Interaction visualization of FT1-3LT0 (b) shows it shares some similar residue interactions with ISO—3LT0 (c).

**Figure 8:**
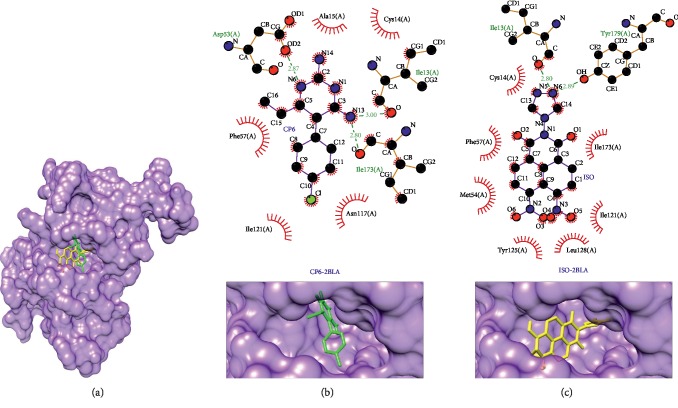
Binding complex and interaction visualization between dihydrofolate reductase, thymidylate synthase (2BLA, dark purple), pyrimethamine (CP6, green), and isoquinoline derivative (ISO, yellow). In the ISO-CP6-2BLA binding complex (a), CP6 and ISO share the same binding pocket. Interaction visualization of CP6-2BLA (b) shows a few similar hydrophobic interactions with ISO-2BLA (c).

Enoyl-acyl carrier protein reductase (PDB ID: 3LT0) and dihydrofolate reductase-thymidylate synthase (PDB ID: 2BLA), as shown in Figures 7 and 8, respectively, have the same difference as a the result of reverse docking, which is −1.8 kcal/mol compared to each native ligand of protein targets. Enoyl-acyl carrier protein reductase plays role in catalyzation of the final reaction in the type II fatty acid synthesize (FAS) system; this protein mediates the reduction of NADH-dependent trans-2-enoyl-ACP (acyl carrier protein) to acyl-ACP [66]. In contrast in humans, this enzyme utilizes type I FAS [67]. The same interaction between this complex protein target-ligand to its native ligand is at Thr266(A) as hydrogen bond and Pro314(A), Gly313(A), Tyr267(A), Tyr111(A), and Leu265(A) as hydrophobic interaction. Ser215(A) and Ala312(A) formed hydrogen bonds in the isoquinoline derivative compound, yet they formed hydrophobic interactions in the native ligand, as shown in Table 5 and Figures 7(b) and 7(c). On the other hand, dihydrofolate reductase-thymidylate synthase has five same interactions: Ile173(A), Cys14(A), Ile121(A), and Phe57(A) formed hydrophobic interactions and Ile13(A) formed hydrogen bonds in the isoquinoline derivative compound. However, both Ile13(A) and Ile173(A) formed the hydrophobic interaction in the native ligand and the rest formed the hydrophobic interaction, as shown in Table 5 and Figures 8(b) and 8(c). This protein target can be inhibited by antifolate agents [23, 64]. In *Plasmodium*, dihydrofolate reductase-thymidylate synthase (DHFR) and thymidylate synthase (TS) have a single bifunctional protein that codes for both DHFR and TS activities. Meanwhile, in mammalian cells, these two proteins have separate genes that code for small monofunctional DHFR and TS. However, it has not been fully understood if this bifunctional status of DHFR-TS in *Plasmodium* can play a role in selective chemotherapy [68].

## 4. Conclusions

On the basis of the results of this research and discussion, it can be concluded that the compound of *Streptomyces hygroscopicus* subsp. hygroscopicus can be identified using LC/MS as 6,7-dinitro-2-[1, 2, 4]triazole-4-yl-benzo[de]isoquinoline-1,3-dione, which is one of the derivatives of isoquinoline. Reverse molecular studies of this compound revealed that this compound has drug-likeness criteria based on Lipinski's rule of five, and thus, it can be administered by the oral route and has antimalarial activity assessed by the strength of binding affinities in each protein target compared to each native ligand using the reverse molecular docking approach. Based on the results, this novel compound can be formulated as an antimalarial drug candidate. The most possible mechanism of action is through the interaction of compound with several key proteins in accordance with their binding affinities; they are as follows: adenylosuccinate synthetase, falcipain 2, glucose-6-phosphate isomerase, glycerol kinase, enoyl-acyl carrier protein reductase, and dihydrofolate reductase-thymidylate synthase. For further study, the compound 3D model should be geometrically optimized, and it should be purified and analyzed further by *in vitro* and *in vivo* analysis.

## Figures and Tables

**Table 1:** The results of protein target screening.

No.		Protein target	PDB ID	Res (Å)	Organism	Native ligand	Percent identity to human protein (accession no.) [17, 18]^*∗*^		Ref.
1.	*Pf*dUTPDXRase	Deoxyuridine 5′-triphosphate nucleotidohydrolase	1VYQ	2.40	*P*. *falciparum*	2,3-Deoxy-3-fluoro-5-O-trityluridine (DUX)	36.59%	1Q5H_A	[16, 19]
2.	*Pf*DHODH	Dihydroorotate dehydrogenase	3O8A	2.30	*P*. *falciparum*	N-Cyclopropyl-5-[2-methyl-5-(trifluoromethoxyl)-1H-benzimidazol-1-yl]thiophene-2-carboxamide (O8A)	38.52%	5K9C_A	[20]
3.	*Pv*DHFR	Dihydrofolate reductase-thymidylate synthase	2BLA	2.50	*P*. *vivax*	Pyrimethamine (CP6)	28.71%	3GHV_A	[21–23]
4.	*Pf*PMT	Phosphoethanolamine N-methyltransferase	4FGZ	1.994	*P*. *falciparum*	Amodiaquine, flavoquine (CQA)	45.83%^†^	3O0Z_A	[16, 24, 25]
5.	*Pf*AdSS	Adenylosuccinate synthetase	1P9B	2.00	*P*. *falciparum*	Hadacidin (HDA)	46.03%	2V40_A	[16, 26, 27]
6.	*Pf*GK	Glycerol kinase	2W41	2.41	*P*. *falciparum*	Adenine diphosphate (ADP)	41.67%^†^	2GY5_A	[16, 28]
7.	GMP synthetase	Guanosine monophosphate synthetase	3UOW	2.72	*P*. *falciparum*	Xanthosine monophosphate (XMP)	30.77%	2VXO_A	[16, 29]
8.	*Pf*ENR	Enoyl-acyl carrier protein reductase	3LT0	1.96	*P*. *falciparum*	4-(2,4-Dichlorophenoxy)-3-hydroxybenzaldehyde (FT1); nicotinamide-adenine-dinucleotide (NAD)	30.00%^‡^	6JLQ_A	[16,30–32]
9.	*Pf*G6PI	Glucose-6-phosphate isomerase	3PR3	2.45	*P*. *falciparum*	Fructose-6-phosphate (F6P)	38.89%	1JLH_A	[16, 33, 34]
10.	*Pf*-cTrpRNA	Tryptophanyl tRNA synthetase	4J75	2.40	*P*. *falciparum*	Tryptophanyl-5′amp (TYM)	50.25%	1ULH_A	[16, 35, 36]
11.	DXR	1-Deoxy-D-xylulose 5-phosphate reductoisomerase	4Y67	1.60	*P*. *falciparum*	[(2R)-2-{2-[Hydroxy(methyl)amino]-2-oxoethyl}-5-phenylpentyl]phosphonic acid (RC5)	28.57%	3FJC_A	[16, 37, 38]
12.	DXR	1-Deoxy-D-xylulose 5-phosphate reductoisomerase	3AU9	1.90	*P*. *falciparum*	3-[Formyl(hydroxyl)amino]propylphosphonic (fosmidomycin/FOM)	28.57%	3FJC_A	[39]
13.	FP2	Falcipain 2	3BPF	2.90	*P*. *falciparum*	N-[N-[1-Hydroxycarboxyethyl-carbonyl]leucylamino-butyl]-guanindine (E64)	38.50%	1FH0_A	[6,40–42]
14.	FP3	Falcipain 3	3BWK	2.42	*P*. *falciparum*	N∼2∼-(Morpholin-4-ylcarbonyl)-N-[(3S)-1-phenyl-5-phenylsulfonyl)pentan-3-yl)]-L-leucinamide (C1P)	78.00%	4AHH_A	[6, 40, 43]

^*∗*^Determined using blast.ncbi.nlm.nih.gov (see supplementary files [Supplementary-material supplementary-material-1] and [Supplementary-material supplementary-material-1]). Percent identity was noted by comparing the sequence of protein targets with the most similar protein sequence existed on humans (accession no. of protein). The Expect value (*E* value) used was default in the BLAST system (<10), except percent with marks (^†^10–100, ^‡^100–1000).

**Table 2:** Grid box scale of reverse molecular docking.

No.	Protein targets (PDB ID)	Control ligand	Grid box scale					
			Center X	Center Y	Center Z	Size X	Size Y	Size Z
1	1VYQ	DUX	39.642	−−11.423	−9.416	20.929	18.116	18.059
2	3O8A	O8A	27.327	−11.500	17.077	19.029	14.315	14.932
3	2BLA	CP6	28.120	3.267	11.793	11.999	13.358	17.359
4	4FGZ	CQA	19.138	26.353	14.428	31.372	25.182	30.762
5	1P9B	HDA	26.315	86.090	28.993	13.599	14.757	14.304
6	2W41	ADP	7.699	−11.466	110.859	13.817	19.345	20.104
7	3UOW	XMP	−10.921	30.878	43.840	22.647	19.535	21.819
8	3LT0	FT1	49.285	86.071	37.026	37.026	29.896	18.603
		NAD						
9	3PR3	F6P	15.645	12.408	12.578	20.050	20.150	19.285
10	4J75	TYM	21.709	12.607	−0.525	21.240	29.604	28.658
11	4Y67	RC5	2.187	12.936	19.733	21.369	18.817	19.252
12	3BPF	E64	−59.109	−3.131	−18.321	25.043	20.228	22.512
13	3BWK	C1P	5.661	−23.586	50.308	24.306	22.568	19.700
14	3AU9	FOM	11.486	−15.360	16.082	20.921	16.018	18.591

**Table 3:** The results of derivative compound identification using LC/MS.

No.	RT	Precursor ion				Possibility name	Product ion			
		Ion formula	*m*/*z*	Err (mDa)	*M* sigma		Ion formula	*m*/*z*	Err (mDa)	*m* sigma
1	2.52	C_7_H_13_N_2_O^1^^*-*^	141.1022	0.1	6.7	—	C_6_H_11_N_2_^1^^+^	111.0917	0.6	8.5
2	3.77	C_14_H_7_N_6_O_6_^1^^+^	355.0422	1.6	6.9	6,7-Dinitro-2-[1, 2, 4]triazole-4-yl-benzo[de]isoquinoline-1,3-dione	C_6_HN_6_O_2_^1^^+^	189.0155	0.9	5.2
							C_8_H_7_O_4_^1^^+^	167.0339	0.2	2.9
3	4.08	C_16_H_19_N_4_O_5_^1^^+^	347.1350	−1.7	−4.9	—	—	—	—	—
4	8.15	C_25_H_40_N_5_O_8_^1^^+^	538.2871	−0.5	6.10	—	C_16_H_22_NO_3_^1^^+^	276.1594	−0.3	17.9
5	9.22	C_22_H_30_NO_6_^1^^+^	404.2100	−1.40	5.80	—	C_22_H_27_NO_6_^1^^+^	387.1802	−1.4	8.8
							C_14_H_19_O_5_^1^^+^	267.1227	0.6	13.1
							C_16_H_24_NO_2_^1^^+^	262.1802	1.7	2.1
							C_14_H_26_NO_2_^1^^+^	240.1958	−0.9	11.4
							C_8_H_9_O^1^^+^	121.0648	0.1	12.1
							C_8_H_9_^1^^+^	105.0699	−0.1	17.6

**Table 4:** Prediction of pharmacokinetic profile.

Molecular weight	354.23 g/mol
Hydrogen bond donor	0
Hydrogen bond acceptor	8
cLogP	0.08

**Table 5:** Interaction between the isoquinoline derivative and the protein target compared to its native ligand.

No.	Protein targets (PDB ID)	Ligand	Interactions
1	1P9B	ISO	Hydrogen bond: Val246(A), **Arg311(A), Arg313(A), Thr307(A), Gly53(A),** Asn232(A)
			Hydrophobic interaction: Thr247(A), Leu236(A), **Thr 141(A), Val281(A), Gly306(A), Thr308(A), Asp26(A),** His54(A), Asn51(A), Ala52(A)
		HDA	Hydrogen bond: **Thr307(A), Gly53(A), Arg313(A),** Thr309(A), **Thr 308(A)**, **Arg311(A), Asp26(A).**
			Hydrophobic interaction: **Gly306(A)**, **Val281(A), Thr141(A)**

2	3BPF	ISO	Hydrogen bond: **Asn77(A),** Ala110(A), Gln208(C)
			Hydrophobic interaction: Ser74(A), Gly79(A), Phe75(A), **Tyr78(A),** Asn81(A), Cys80(A), Asp109(A), Asn112(A), Pro111(A), Asp72(A), Val171(A)
		E64	Hydrogen bond: Lys76(A), **Asn77(A),** Asn87(A), Gly83(A),
			Hydrophobic interaction: Leu84(A), Asp234(A), Ile85(A), **Tyr78(A),** Ser149(A), Gly82(A), Asn173(A), Ala175(A), His174(A), Cys42(A)

3	3PR3	ISO	Hydrogen bond: Lys540(A), Arg91(A), **Gly293(A), Lys232(A), Gln533(A)**
			Hydrophobic interaction: **Thr236(A),** Ile156(A), **Thr233(A),** Gly292(A), **Gln376(A), Arg294(A), Glu380(A)**
		F6P	Hydrogen bond: **Glu380(A), Arg294(A), Thr233(A),** Ser231(A), **Thr236(A**), Ser159(A), **Lys232(A)**
			Hydrophobic interaction: **Gln533(A), Gly293(A), Gln376(A)**

4	2W41	ISO	Hydrogen bond: Ser317(A), **Thr268(A), Ser332(A)**
			Hydrophobic interaction: **Val316(A)**, **Gly313(A)**, **Pro328(A),** Ser329(A), **Gly414(A)**, **Asn418(A), Lys417(A), Met415(A)**
		ADP	Hydrogen bond: **Asn418(A), Ser332(A)**, **Pro328(A)**
			Hydrophobic interaction: **Thr268(A), Gly313(A),** Gly267(A), **Val316(A), Gly414(A)**, **Lys417(A)**, Ala331(A), **Met415(A)**

5	3LT0	ISO	Hydrogen bond: Gly104(A), **Thr266(A), Ser215(A), Ala312(A),** Ser317(A)
			Hydrophobic interaction: Leu216(A), Ala319(A), Ala320(A), **Pro314(A), Gly313(A)**, **Tyr267(A), Tyr111(A)**, **Leu265(A)**
		FT1	Hydrogen bond: **Thr266(A)**
			Hydrophobic interaction: **Tyr111(A), Ser215(A), Pro314(A), Gly313(A), Ala312(A), Tyr267(A),** Tyr277(A), **Leu265(A)**

6	2BLA	ISO	Hydrogen bond: Tyr179(A), **Ile13(A)**
			Hydrophobic interaction: **Ile173(A), Cys14(A), Phe57(A)**, Met54(A), Leu128(A), Tyr125(A), **Ile121(A)**
		CP6	Hydrogen bond: **Ile13(A),** Asp53(A), **Ile173(A)**
			Hydrophobic interaction: Ala15(A), **Cys14(A)**, **Phe57(A), Ile121(A),** Asn117(A)

Protein targets: adenylosuccinate synthetase (1P9B), falcipain 2 (3BPF), glucose-6-phosphate isomerase (3PR3), glycerol kinase (2W41), enoyl-acyl carrier protein reductase (3LT0), and dihydrofolate reductase-thymidylate synthase (2BLA). Ligands: 6,7-dinitro-2-[1, 2, 4]triazol-4-yl-benzo[de]isoquinoline-1,3-dione (ISO), hadacidin (HDA), hydroxycarboxyethyl-carbonyl [leucylamino-butyl]-guanindine (E64), fructose-6-phosphate (F6P), adenine diphosphate (ADP), 4-(2,4-dichlorophenoxy)-3-hydroxybenzaldehyde (FT1), and pyrimethamine (CP6).

## Data Availability

The protein structures can be accessed with the PDB codes 1VYQ, 3O8A, 2BLA, 4FGZ, 1P9B, 2W41, 3UOW, 3LT0, 3PR3, 4J75, 4Y67, 3BPF, and 3BWK, and the native ligands can be accessed in PubChem.

## Supplementary Materials

blast.ncbi.nlm.nih.gov blast.ncbi.nlm.nih.gov Supplementary Materials Supplementary 1 (S1): BLAST results of protein targets used (determined using blast.ncbi.nlm.nih.gov). Percent of identity was noted by comparing the sequence of protein targets with the most similar protein sequence existed on human (accession no. of protein). The Expect value (*E* value) used was default in the BLAST system. Supplementary 2 (S2): distance tree results of the protein targets acquired from BLAST analysis (determined using blast.ncbi.nlm.nih.gov).
