# Supplementary material for: Antimalarial Properties of Isoquinoline Derivative from Streptomyces hygroscopicus subsp. Hygroscopicus: An In Silico Approach
Source: Biomed Res Int. 2020 Jan 8;2020:6135696. doi: 10.1155/2020/6135696 (PMC6973190; doi:10.1155/2020/6135696)
Supplement: blast.ncbi.nlm.nih.gov blast.ncbi.nlm.nih.gov Supplementary Materials — Supplementary 1 (S1): BLAST results of protein targets used (determined using blast.ncbi.nlm.nih.gov). Percent of identity was noted by comparing the sequence of protein targets with the most similar protein sequence existed on human (accession no. of protein). The Expect value (E value) used was default in the BLAST system. Supplementary 2 (S2): distance tree results of the protein targets acquired from BLAST analysis (determined using blast.ncbi.nlm.nih.gov). [file 6135696.f1.zip › 6135696.f1/S2.Distance Tree of Results of Target Proteins.pdf]

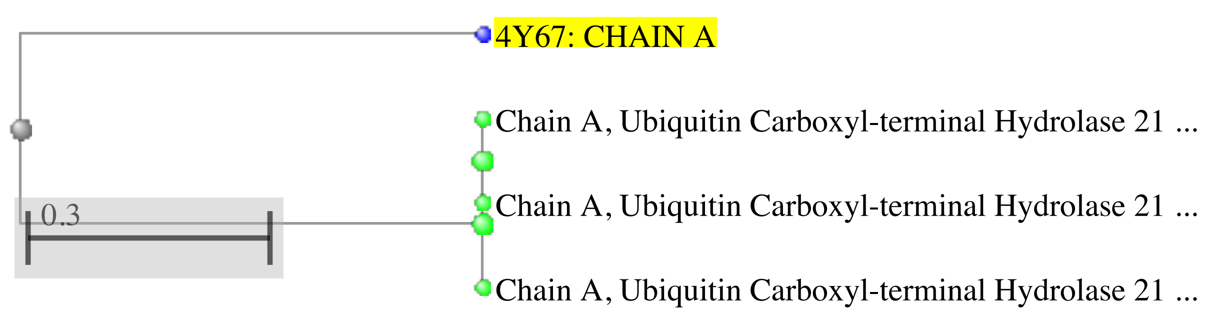
4Y67: CHAIN A
Chain A, Ubiquitin Carboxyl-terminal Hydrolase 21 ...
Chain A, Ubiquitin Carboxyl-terminal Hydrolase 21 ...
Chain A, Ubiquitin Carboxyl-terminal Hydrolase 21 ...
0.3

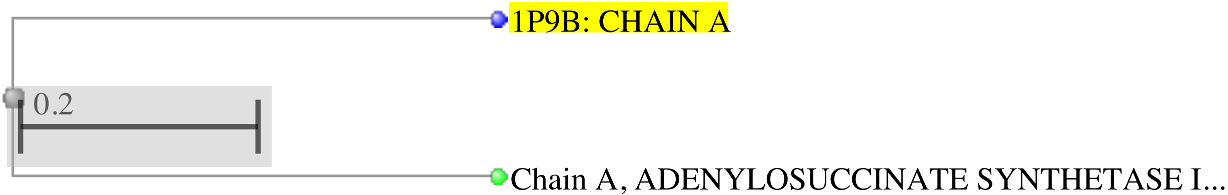
1P9B: CHAIN A
0.2
Chain A, ADENYLOSUCCINATE SYNTHETASE I...

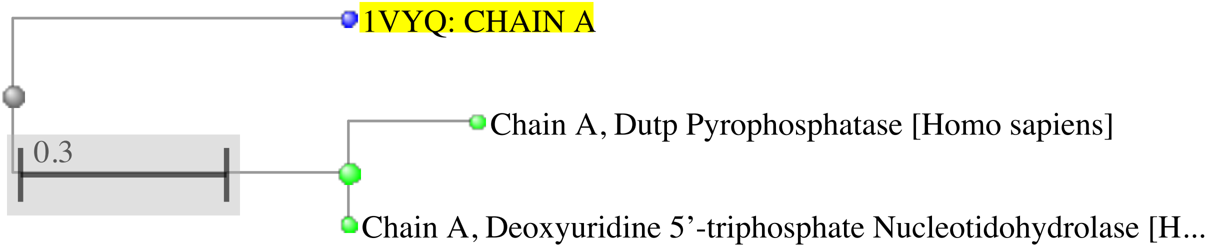
1VYQ: CHAIN A
Chain A, Dutp Pyrophosphatase [Homo sapiens]
0.3
Chain A, Deoxyuridine 5'-triphosphate Nucleotidohydrolase [H...

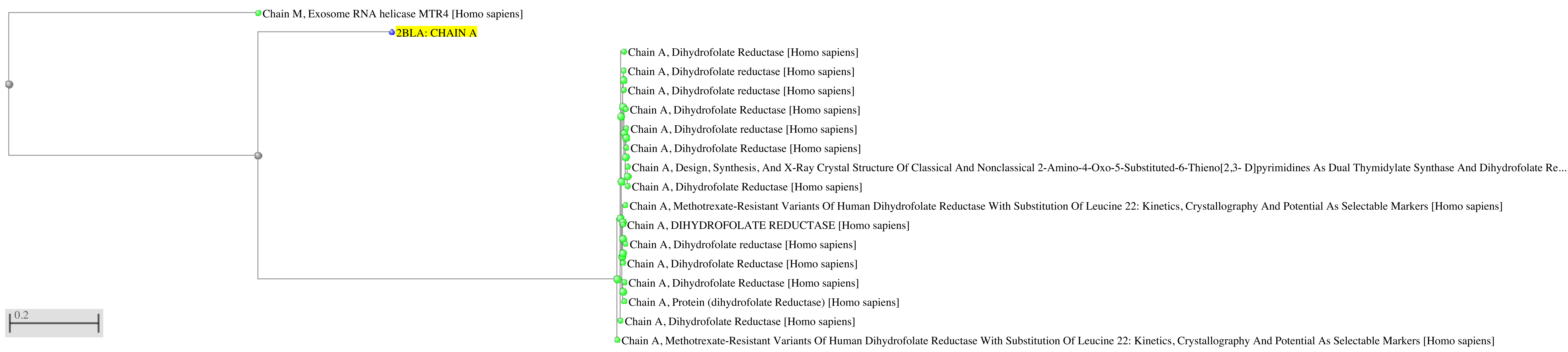

Chain M, Exosome RNA helicase MTR4 [Homo sapiens]
2BLA: CHAIN A
Chain A, Dihydrofolate Reductase [Homo sapiens]
Chain A, Dihydrofolate reductase [Homo sapiens]
Chain A, Dihydrofolate reductase [Homo sapiens]
Chain A, Dihydrofolate Reductase [Homo sapiens]
Chain A, Dihydrofolate reductase [Homo sapiens]
Chain A, Dihydrofolate Reductase [Homo sapiens]
Chain A, Design, Synthesis, And X-Ray Crystal Structure Of Classical And Nonclassical 2-Amino-4-Oxo-5-Substituted-6-Thieno[2,3- D]pyrimidines As Dual Thymidylate Synthase And Dihydrofolate Re...
Chain A, Dihydrofolate Reductase [Homo sapiens]
Chain A, Methotrexate-Resistant Variants Of Human Dihydrofolate Reductase With Substitution Of Leucine 22: Kinetics, Crystallography And Potential As Selectable Markers [Homo sapiens]
Chain A, DIHYDROFOLATE REDUCTASE [Homo sapiens]
Chain A, Dihydrofolate reductase [Homo sapiens]
Chain A, Dihydrofolate Reductase [Homo sapiens]
Chain A, Dihydrofolate Reductase [Homo sapiens]
Chain A, Protein (dihydrofolate Reductase) [Homo sapiens]
Chain A, Dihydrofolate Reductase [Homo sapiens]
Chain A, Methotrexate-Resistant Variants Of Human Dihydrofolate Reductase With Substitution Of Leucine 22: Kinetics, Crystallography And Potential As Selectable Markers [Homo sapiens]
0.2

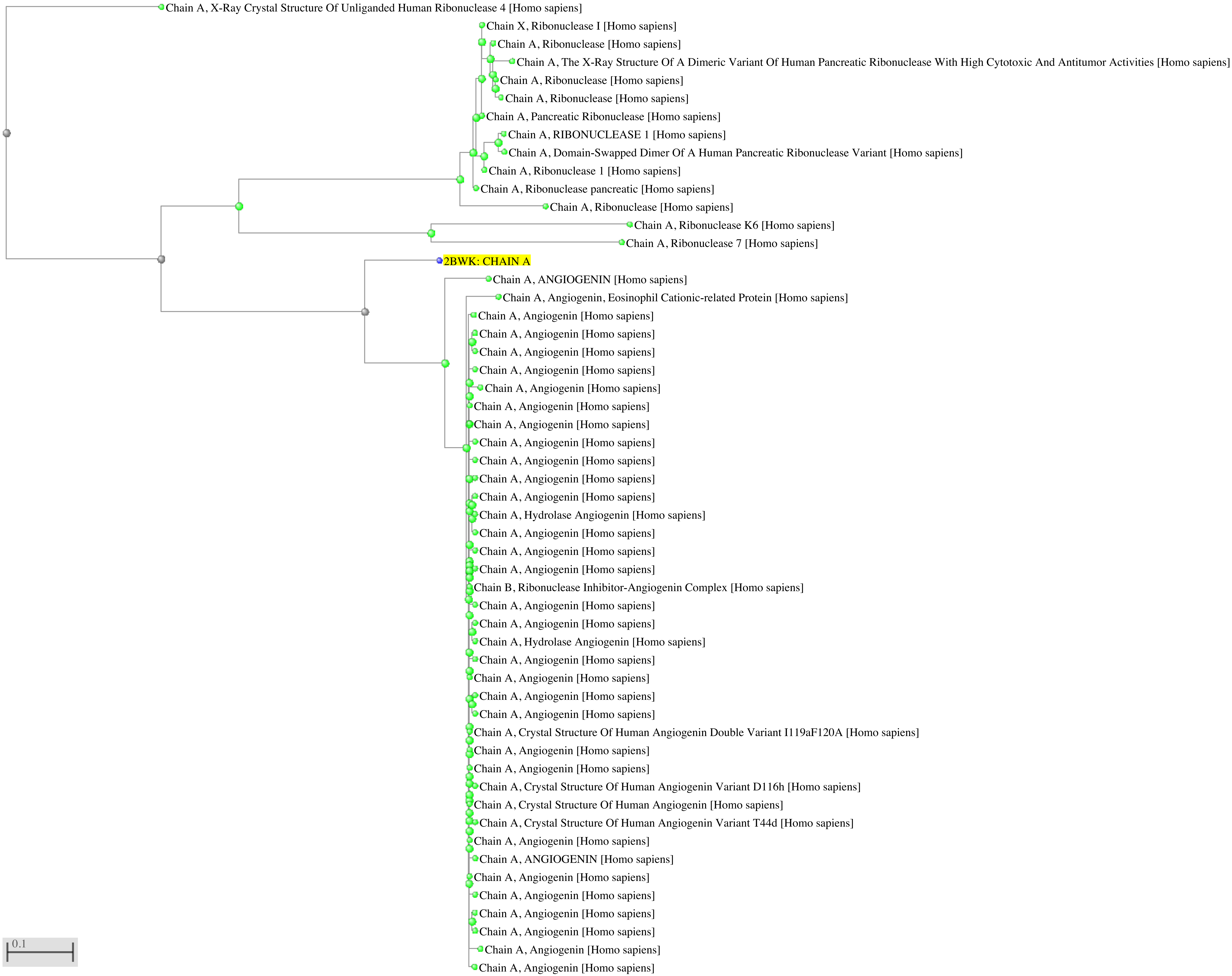
Chain A, X-Ray Crystal Structure Of Unliganded Human Ribonuclease 4 [Homo sapiens]
Chain X, Ribonuclease I [Homo sapiens]
Chain A, Ribonuclease [Homo sapiens]
Chain A, The X-Ray Structure Of A Dimeric Variant Of Human Pancreatic Ribonuclease With High Cytotoxic And Antitumor Activities [Homo sapiens]
Chain A, Ribonuclease [Homo sapiens]
Chain A, Ribonuclease [Homo sapiens]
Chain A, Pancreatic Ribonuclease [Homo sapiens]
Chain A, RIBONUCLEASE 1 [Homo sapiens]
Chain A, Domain-Swapped Dimer Of A Human Pancreatic Ribonuclease Variant [Homo sapiens]
Chain A, Ribonuclease 1 [Homo sapiens]
Chain A, Ribonuclease pancreatic [Homo sapiens]
Chain A, Ribonuclease [Homo sapiens]
Chain A, Ribonuclease K6 [Homo sapiens]
Chain A, Ribonuclease 7 [Homo sapiens]
2BWK: CHAIN A
Chain A, ANGIOGENIN [Homo sapiens]
Chain A, Angiogenin, Eosinophil Cationic-related Protein [Homo sapiens]
Chain A, Angiogenin [Homo sapiens]
Chain A, Angiogenin [Homo sapiens]
Chain A, Angiogenin [Homo sapiens]
Chain A, Angiogenin [Homo sapiens]
Chain A, Angiogenin [Homo sapiens]
Chain A, Angiogenin [Homo sapiens]
Chain A, Angiogenin [Homo sapiens]
Chain A, Angiogenin [Homo sapiens]
Chain A, Angiogenin [Homo sapiens]
Chain A, Angiogenin [Homo sapiens]
Chain A, Angiogenin [Homo sapiens]
Chain A, Hydrolase Angiogenin [Homo sapiens]
Chain A, Angiogenin [Homo sapiens]
Chain A, Angiogenin [Homo sapiens]
Chain A, Angiogenin [Homo sapiens]
Chain B, Ribonuclease Inhibitor-Angiogenin Complex [Homo sapiens]
Chain A, Angiogenin [Homo sapiens]
Chain A, Angiogenin [Homo sapiens]
Chain A, Hydrolase Angiogenin [Homo sapiens]
Chain A, Angiogenin [Homo sapiens]
Chain A, Angiogenin [Homo sapiens]
Chain A, Angiogenin [Homo sapiens]
Chain A, Angiogenin [Homo sapiens]
Chain A, Crystal Structure Of Human Angiogenin Double Variant I119aF120A [Homo sapiens]
Chain A, Angiogenin [Homo sapiens]
Chain A, Angiogenin [Homo sapiens]
Chain A, Crystal Structure Of Human Angiogenin Variant D116h [Homo sapiens]
Chain A, Crystal Structure Of Human Angiogenin [Homo sapiens]
Chain A, Crystal Structure Of Human Angiogenin Variant T44d [Homo sapiens]
Chain A, Angiogenin [Homo sapiens]
Chain A, ANGIOGENIN [Homo sapiens]
Chain A, Angiogenin [Homo sapiens]
Chain A, Angiogenin [Homo sapiens]
Chain A, Angiogenin [Homo sapiens]
Chain A, Angiogenin [Homo sapiens]
Chain A, Angiogenin [Homo sapiens]
Chain A, Angiogenin [Homo sapiens]
0.1

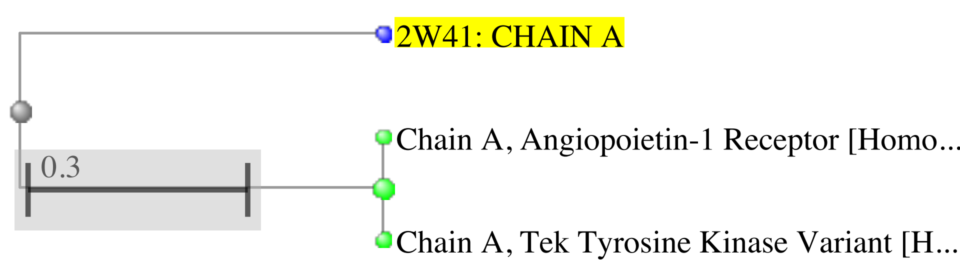
2W41: CHAIN A
0.3
Chain A, Angiopoietin-1 Receptor [Homo...
Chain A, Tek Tyrosine Kinase Variant [H...

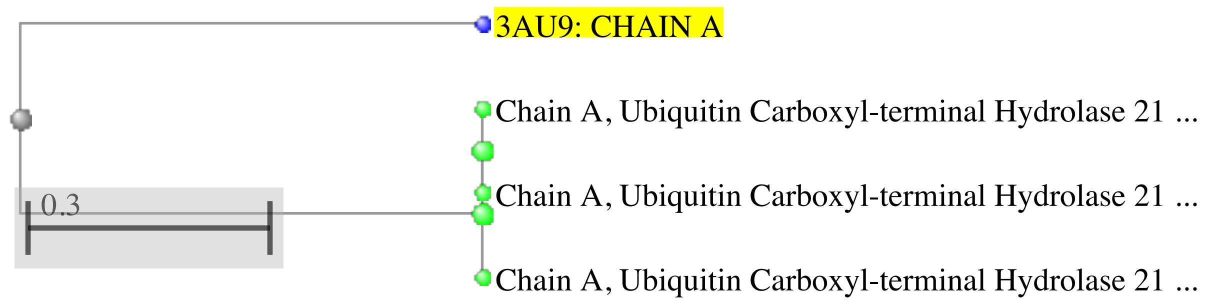
3AU9: CHAIN A
Chain A, Ubiquitin Carboxyl-terminal Hydrolase 21 ...
Chain A, Ubiquitin Carboxyl-terminal Hydrolase 21 ...
Chain A, Ubiquitin Carboxyl-terminal Hydrolase 21 ...
0.3

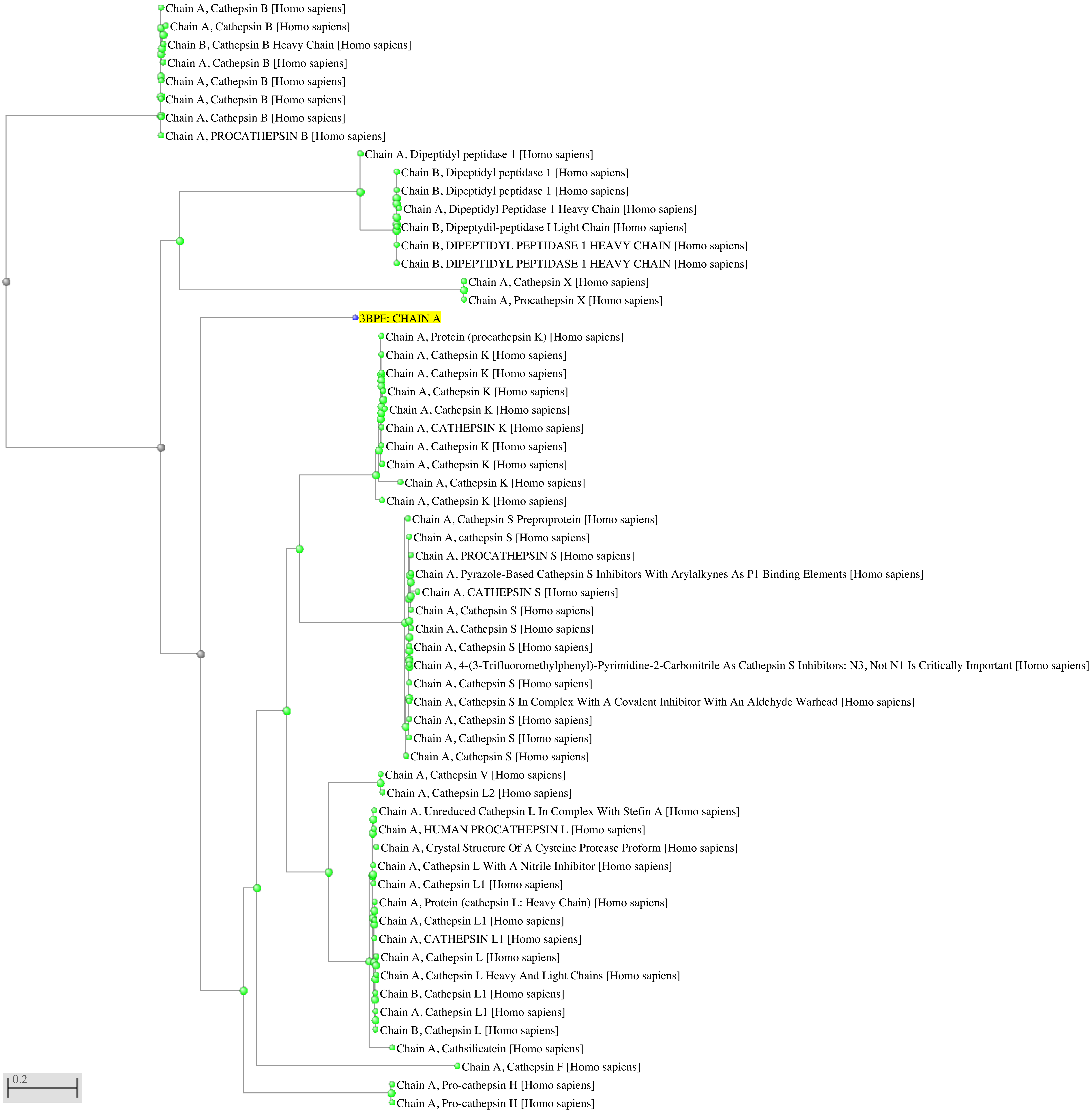
Chain A, Cathepsin B [Homo sapiens]
Chain A, Cathepsin B [Homo sapiens]
Chain B, Cathepsin B Heavy Chain [Homo sapiens]
Chain A, Cathepsin B [Homo sapiens]
Chain A, Cathepsin B [Homo sapiens]
Chain A, Cathepsin B [Homo sapiens]
Chain A, Cathepsin B [Homo sapiens]
Chain A, PROCATHEPSIN B [Homo sapiens]
Chain A, Dipeptidyl peptidase 1 [Homo sapiens]
Chain B, Dipeptidyl peptidase 1 [Homo sapiens]
Chain B, Dipeptidyl peptidase 1 [Homo sapiens]
Chain A, Dipeptidyl Peptidase 1 Heavy Chain [Homo sapiens]
Chain B, Dipeptydil-peptidase I Light Chain [Homo sapiens]
Chain B, DIPEPTIDYL PEPTIDASE 1 HEAVY CHAIN [Homo sapiens]
Chain B, DIPEPTIDYL PEPTIDASE 1 HEAVY CHAIN [Homo sapiens]
Chain A, Cathepsin X [Homo sapiens]
Chain A, Procathepsin X [Homo sapiens]
3BPF: CHAIN A
Chain A, Protein (procathepsin K) [Homo sapiens]
Chain A, Cathepsin K [Homo sapiens]
Chain A, Cathepsin K [Homo sapiens]
Chain A, Cathepsin K [Homo sapiens]
Chain A, Cathepsin K [Homo sapiens]
Chain A, CATHEPSIN K [Homo sapiens]
Chain A, Cathepsin K [Homo sapiens]
Chain A, Cathepsin K [Homo sapiens]
Chain A, Cathepsin K [Homo sapiens]
Chain A, Cathepsin K [Homo sapiens]
Chain A, Cathepsin S Preproprotein [Homo sapiens]
Chain A, cathepsin S [Homo sapiens]
Chain A, PROCATHEPSIN S [Homo sapiens]
Chain A, Pyrazole-Based Cathepsin S Inhibitors With Arylalkynes As P1 Binding Elements [Homo sapiens]
Chain A, CATHEPSIN S [Homo sapiens]
Chain A, Cathepsin S [Homo sapiens]
Chain A, Cathepsin S [Homo sapiens]
Chain A, Cathepsin S [Homo sapiens]
Chain A, 4-(3-Trifluoromethylphenyl)-Pyrimidine-2-Carbonitrile As Cathepsin S Inhibitors: N3, Not N1 Is Critically Important [Homo sapiens]
Chain A, Cathepsin S [Homo sapiens]
Chain A, Cathepsin S In Complex With A Covalent Inhibitor With An Aldehyde Warhead [Homo sapiens]
Chain A, Cathepsin S [Homo sapiens]
Chain A, Cathepsin S [Homo sapiens]
Chain A, Cathepsin S [Homo sapiens]
Chain A, Cathepsin V [Homo sapiens]
Chain A, Cathepsin L2 [Homo sapiens]
Chain A, Unreduced Cathepsin L In Complex With Stefin A [Homo sapiens]
Chain A, HUMAN PROCATHEPSIN L [Homo sapiens]
Chain A, Crystal Structure Of A Cysteine Protease Proform [Homo sapiens]
Chain A, Cathepsin L With A Nitrile Inhibitor [Homo sapiens]
Chain A, Cathepsin L1 [Homo sapiens]
Chain A, Protein (cathepsin L: Heavy Chain) [Homo sapiens]
Chain A, Cathepsin L1 [Homo sapiens]
Chain A, CATHEPSIN L1 [Homo sapiens]
Chain A, Cathepsin L [Homo sapiens]
Chain A, Cathepsin L Heavy And Light Chains [Homo sapiens]
Chain B, Cathepsin L1 [Homo sapiens]
Chain A, Cathepsin L1 [Homo sapiens]
Chain B, Cathepsin L [Homo sapiens]
Chain A, Cathsilicatein [Homo sapiens]
Chain A, Cathepsin F [Homo sapiens]
Chain A, Pro-cathepsin H [Homo sapiens]
Chain A, Pro-cathepsin H [Homo sapiens]
0.2

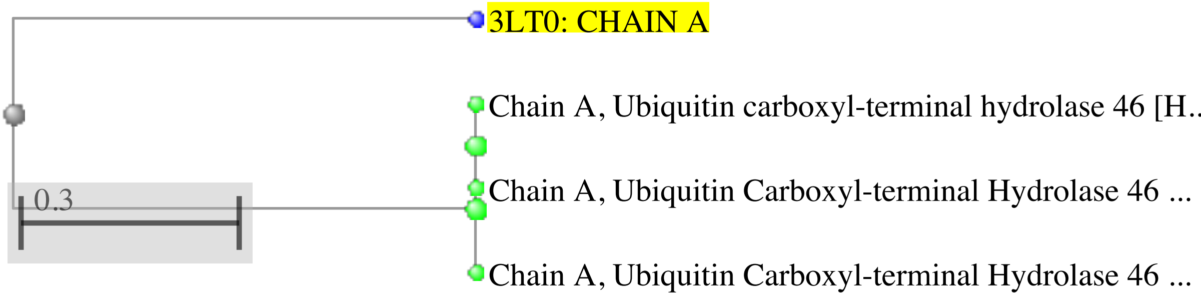
3LT0: CHAIN A
Chain A, Ubiquitin carboxyl-terminal hydrolase 46 [H..
Chain A, Ubiquitin Carboxyl-terminal Hydrolase 46 ...
Chain A, Ubiquitin Carboxyl-terminal Hydrolase 46 ...
0.3

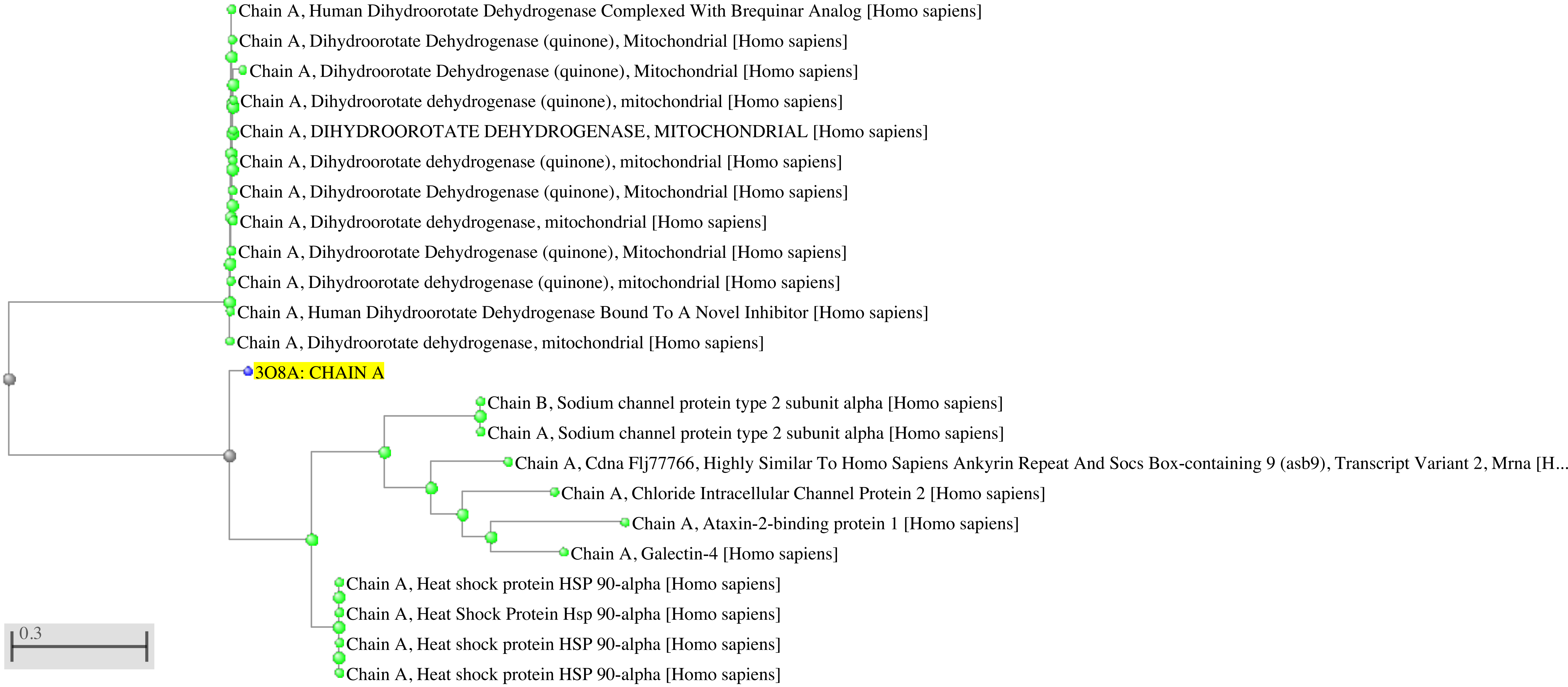
Chain A, Human Dihydroorotate Dehydrogenase Complexed With Brequinar Analog [Homo sapiens]
Chain A, Dihydroorotate Dehydrogenase (quinone), Mitochondrial [Homo sapiens]
Chain A, Dihydroorotate Dehydrogenase (quinone), Mitochondrial [Homo sapiens]
Chain A, Dihydroorotate dehydrogenase (quinone), mitochondrial [Homo sapiens]
Chain A, DIHYDROOROTATE DEHYDROGENASE, MITOCHONDRIAL [Homo sapiens]
Chain A, Dihydroorotate dehydrogenase (quinone), mitochondrial [Homo sapiens]
Chain A, Dihydroorotate Dehydrogenase (quinone), Mitochondrial [Homo sapiens]
Chain A, Dihydroorotate dehydrogenase, mitochondrial [Homo sapiens]
Chain A, Dihydroorotate Dehydrogenase (quinone), Mitochondrial [Homo sapiens]
Chain A, Dihydroorotate dehydrogenase (quinone), mitochondrial [Homo sapiens]
Chain A, Human Dihydroorotate Dehydrogenase Bound To A Novel Inhibitor [Homo sapiens]
Chain A, Dihydroorotate dehydrogenase, mitochondrial [Homo sapiens]
3O8A: CHAIN A
Chain B, Sodium channel protein type 2 subunit alpha [Homo sapiens]
Chain A, Sodium channel protein type 2 subunit alpha [Homo sapiens]
Chain A, Cdna Flj77766, Highly Similar To Homo Sapiens Ankyrin Repeat And Socs Box-containing 9 (asb9), Transcript Variant 2, Mrna [H...
Chain A, Chloride Intracellular Channel Protein 2 [Homo sapiens]
Chain A, Ataxin-2-binding protein 1 [Homo sapiens]
Chain A, Galectin-4 [Homo sapiens]
Chain A, Heat shock protein HSP 90-alpha [Homo sapiens]
Chain A, Heat Shock Protein Hsp 90-alpha [Homo sapiens]
Chain A, Heat shock protein HSP 90-alpha [Homo sapiens]
Chain A, Heat shock protein HSP 90-alpha [Homo sapiens]
0.3

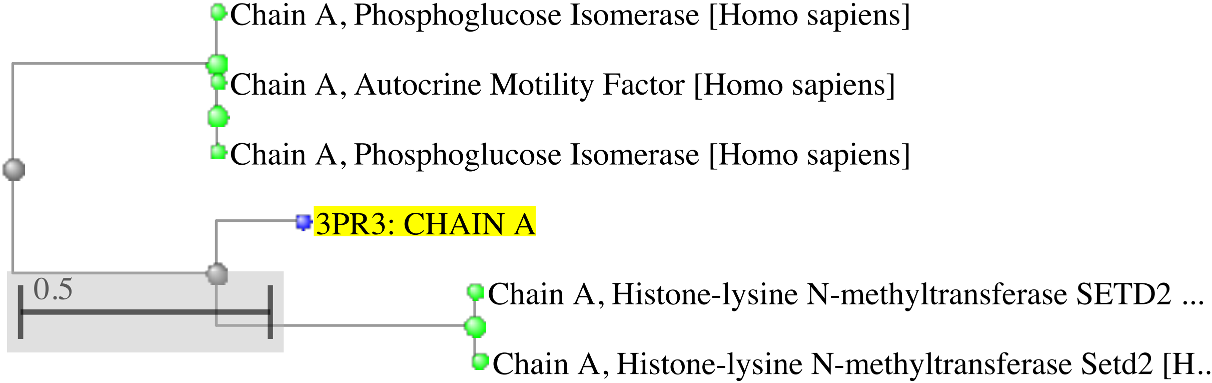
Chain A, Phosphoglucose Isomerase [Homo sapiens]
Chain A, Autocrine Motility Factor [Homo sapiens]
Chain A, Phosphoglucose Isomerase [Homo sapiens]
3PR3: CHAIN A
0.5
Chain A, Histone-lysine N-methyltransferase SETD2 ...
Chain A, Histone-lysine N-methyltransferase Setd2 [H..

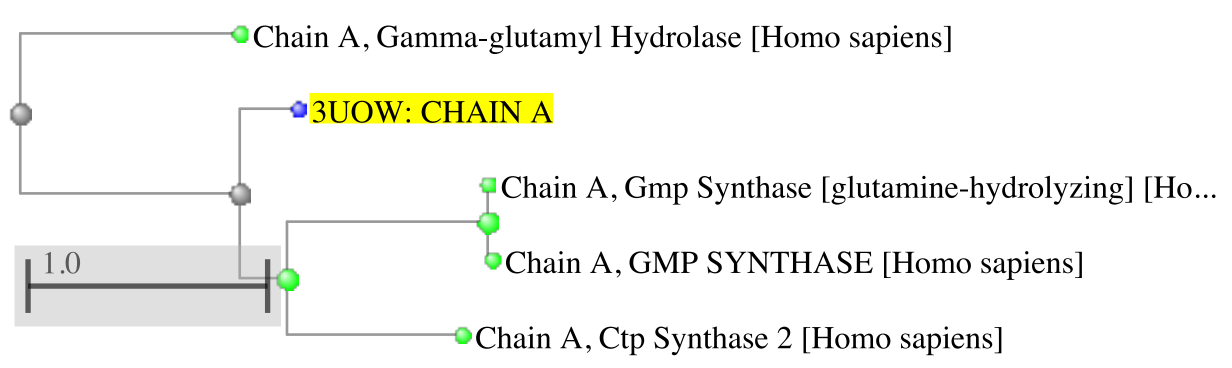
Chain A, Gamma-glutamyl Hydrolase [Homo sapiens]
3UOW: CHAIN A
Chain A, Gmp Synthase [glutamine-hydrolyzing] [Ho...
Chain A, GMP SYNTHASE [Homo sapiens]
Chain A, Ctp Synthase 2 [Homo sapiens]
1.0

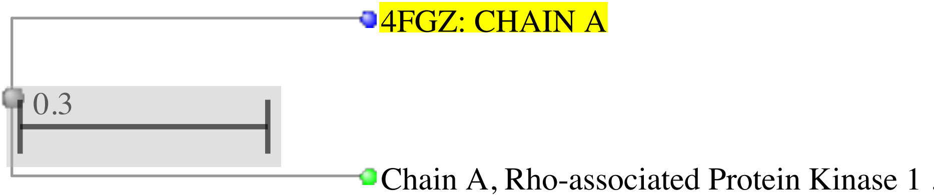
4FGZ: CHAIN A
0.3
Chain A, Rho-associated Protein Kinase 1

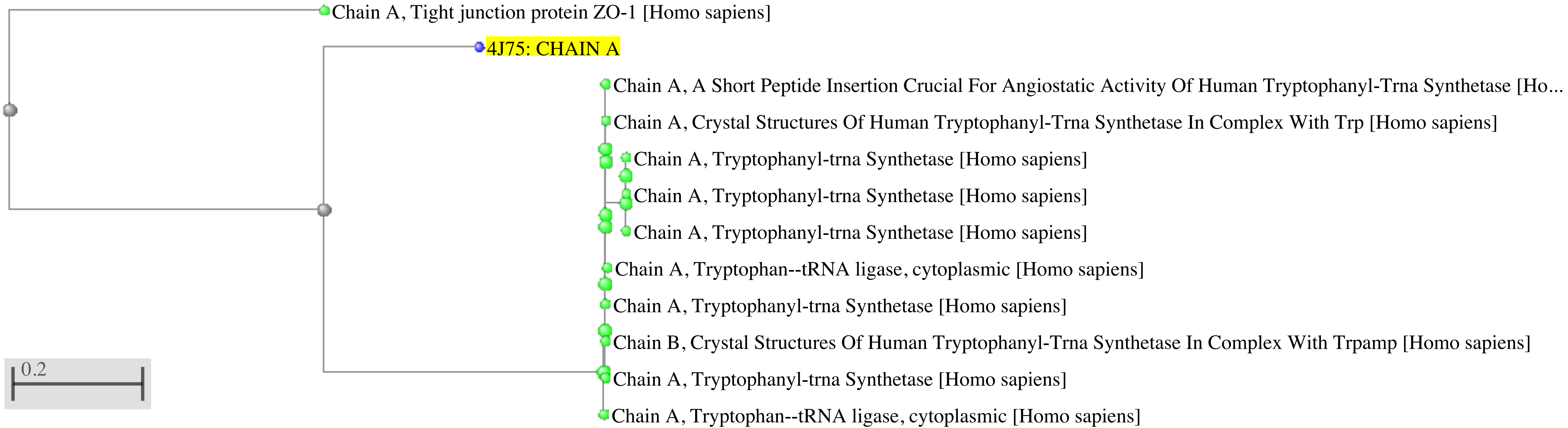
Chain A, Tight junction protein ZO-1 [Homo sapiens]
4J75: CHAIN A
Chain A, A Short Peptide Insertion Crucial For Angiostatic Activity Of Human Tryptophanyl-Trna Synthetase [Ho...
Chain A, Crystal Structures Of Human Tryptophanyl-Trna Synthetase In Complex With Trp [Homo sapiens]
Chain A, Tryptophanyl-trna Synthetase [Homo sapiens]
Chain A, Tryptophanyl-trna Synthetase [Homo sapiens]
Chain A, Tryptophanyl-trna Synthetase [Homo sapiens]
Chain A, Tryptophan--tRNA ligase, cytoplasmic [Homo sapiens]
Chain A, Tryptophanyl-trna Synthetase [Homo sapiens]
Chain B, Crystal Structures Of Human Tryptophanyl-Trna Synthetase In Complex With Trpamp [Homo sapiens]
Chain A, Tryptophanyl-trna Synthetase [Homo sapiens]
Chain A, Tryptophan--tRNA ligase, cytoplasmic [Homo sapiens]
0.2
